# Editorial: Translational applications of neuroimaging

**DOI:** 10.3389/fnins.2024.1400383

**Published:** 2024-03-26

**Authors:** Amelie Haugg, David M. A. Mehler, Stavros Skouras

**Affiliations:** ^1^Department of Child and Adolescent Psychiatry, Psychiatric Hospital, University of Zürich, Zürich, Switzerland; ^2^Department of Psychiatry, Psychotherapy and Psychosomatics, Faculty of Medicine, RWTH Aachen University, Aachen, Germany; ^3^Institute for Translational Psychiatry, University of Münster, Münster, Germany; ^4^Cardiff University Brain Research Imaging Centre, School of Psychology, Cardiff University, Cardiff, United Kingdom; ^5^Department of Fundamental Neurosciences, Faculty of Medicine, University of Geneva, Geneva, Switzerland

**Keywords:** translational applications, neuroimaging, psychiatry, neurology, EEG, MRI, CT, multimodal neuroimaging

Despite substantial progress in the development of neuroimaging methodologies, translational applications of neuroimaging remain scarce. This Research Topic focuses on the latest efforts to transform scientific insights into clinical solutions, highlighting a wide range of promising neuroimaging applications and techniques aimed at improving health outcomes.

Five original studies featured here investigated the use of electroencephalography (EEG) to identify potential neurophysiological biomarkers for the diagnosis and prognosis of neurological and psychiatric disorders. Rockholt et al. conducted a narrative literature review to explore how EEG combined with machine learning algorithms can be utilized as a tool for investigating the neural underpinnings of chronic pain. They underscored the advantages of EEG for studying chronic pain and highlighted the role of machine learning in developing possible biomarkers for this condition. The authors concluded that, in combination with the acquisition of large-scale datasets through collaborative efforts, EEG holds promise for providing valuable information for chronic pain diagnostics in the future. Kenefati et al. empirically assessed the association between EEG oscillations and altered acute pain in chronic pain patients. They observed that chronic pain enhanced pain perception in both pain-affected and pain-unaffected body sites and that distinct brain regions and oscillations were involved in localized hypersensitivity and generalized hyperalgesia. Tao et al. investigated the potential of a limited frontoparietal EEG montage to predict favorable or unfavorable outcomes for comatose patients. Using a retrospective analysis, the authors observed a significant association between a suppressed phase-lag index derived from electrodes F3 and P4 and an unfavorable coma outcome for both stroke patients and patients with traumatic brain injury, demonstrating the value of a limited frontoparietal EEG montage for prognosing coma outcomes. Wang et al. demonstrated how the power of convolutional neural network optimization techniques can be harnessed toward the development of automatic epileptic seizure detection systems for clinical applications. Lastly, Carrle et al. addressed the ubiquitous data science challenge of limited training data in the context of classifying patients suffering from major depressive disorder (MDD) vs. healthy controls using EEG resting-state. First, the authors systematically reviewed existing EEG literature for data augmentation techniques and found reports of classification accuracy improvements ranging between 1 and 40%. They then used data augmentation to generate EEG time-series based on two publicly available case-control EEG data sets that both contained recordings obtained from MDD patients and healthy controls. Their results yielded an improved classification accuracy of up to 10% for one of the two case-control data sets. The authors discuss important challenges worth considering for data augmentation in MDD and other clinical contexts.

Another area with translational potential for neuroimaging is concerned with monitoring changes in brain structure following disease or interventions. Molinski et al. used deep learning to detect hypoxic ischemic encephalopathy after cardiac arrest based on computer tomography (CT) images. Their overall sample comprised 168 CT images, of which about half (52.4%) showed radiological signs, according to expert ratings that were used as ground truth labels. Classification performance on a testing set of 34 independent images revealed accuracies of ~79% with an area under the curve of 93–94%. The authors further provided data that helped interpret the machine learning process by visualizing image features that contributed to the classifier's decision. Further, Shao et al. provide a clinical review emphasizing the role of diffusion tensor imaging for studying the arcuate fasciculus, a key white matter tract implicated in language function. The authors highlight its relevance for exploring potential targets for interventions, as well as for assessing therapeutic progress from neural plasticity, following major brain injuries due to stroke or tumors.

Multimodal brain imaging approaches allow for combining strengths of individual brain imaging techniques and partly overcoming limitations of single modality approaches. Reviews help aggregate such knowledge and highlight new insights that complementary imaging modalities provide for a research field or use case of interest. Korivand et al. contributed a systematic review of studies on the neural correlates of human locomotion. EEG was the most commonly used neuroimaging technique to study locomotion, followed by fMRI, functional near-infrared spectroscopy (fNIRS), and positron emission tomography (PET). Ecologically valid tasks, such as cycling and walking in real-world settings are noted as particularly promising for translational purposes. A key insight is that neural correlates of locomotion have thus far mostly been in healthy individuals. The review concludes with recommendations for the future development of the locomotion imaging research field. Konrad et al. performed a review of neurostimulation and neuroimaging methods used to promote Interpersonal Neural Synchrony, which is considered a key facilitator of empathy, emotion regulation, and prosocial commitment. The review highlights the potential of hyperscanning to investigate neural mechanisms of impaired social interactions in Autism Spectrum Disorder (ASD), Reactive Attachment Disorder (RAD), and Social Anxiety Disorder (SAD). Current limitations and future steps toward clinical utility are presented, including a potential role for combining hyperscanning and neurofeedback (hyperfeedback) as a potential new intervention to target impaired social interactions.

Particularly, the use of real-time fMRI neurofeedback (rt-fMRI-NFB) is emerging as a promising neuroimaging-based intervention technique aiming to normalize dysfunctional brain signals associated with clinical symptoms. With an original rt-fMRI-NFB study, Lieberman et al. tested the feasibility of downregulating activity in the amygdala or the posterior cingulate cortex (PCC) in a sample of patients suffering from post-traumatic stress disorder (PTSD). Both groups showed similar downregulation performance. However, compared to the amygdala downregulation group, patients in the PCC group showed decreased neural activity in areas associated with PTSD psychopathology, including higher visual cortices as well as temporoparietal regions. Behavioral data indicated that only the PCC group showed improvements in PTSD symptoms, suggesting that observed network changes following PCC downregulation may have therapeutic effects. Watve et al. investigated the feasibility of a rt-fMRI-NFB application that coupled ongoing amygdala activity with a visual feedback display of dynamic emotional faces. Healthy participants, divided into four groups, were instructed to either upregulate or downregulate their amygdala activity based on feedback ranging from either fearful to neutral or neutral to happy faces. Amygdala activity in groups receiving feedback based on fearful faces, but not happy faces, showed signal modulations. Further, valence-dependent changes in effective connectivity between the fusiform face area and the amygdala were observed. However, neurofeedback training did not lead to changes in clinical and behavioral measures.

Concluding, this Research Topic draws together diverse findings from the latest research across multiple neuroimaging modalities and clinical use cases. According to this, particularly promising near-future applications of translational neuroimaging include the use of machine learning approaches and real-time interventions in the management of chronic and acute pain, epilepsy, encephalopathy, coma, stroke, MDD, PTSD, ASD, RAD, and SAD.

## Author contributions

AH: Writing – original draft, Writing – review & editing. DM: Writing – original draft, Writing – review & editing. SS: Writing – original draft, Writing – review & editing.

